# African Swine Fever Outbreaks in Lithuanian Domestic Pigs in 2019

**DOI:** 10.3390/ani12010115

**Published:** 2022-01-04

**Authors:** Alvydas Malakauskas, Katja Schulz, Indrė Kukanauskaitė, Marius Masiulis, Franz Josef Conraths, Carola Sauter-Louis

**Affiliations:** 1 Department of Veterinary Pathobiology, Veterinary Academy, Lithuanian University of Health Sciences, Tilžės 18, LT44307 Kaunas, Lithuania; indre.kukanauskaite@vmvt.lt; 2 State Food and Veterinary Service, Siesiku 19, LT07170 Vilnius, Lithuania; marius.masiulis@nmvrvi.lt; 3 Friedrich-Loeffler-Institut, Federal Research Institute for Animal Health, Institute of Epidemiology, Südufer 10, 17493 Greifswald-Insel Riems, Germany; katja.schulz@fli.de (K.S.); franz.conraths@fli.de (F.J.C.); carola.sauter-louis@fli.de (C.S.-L.); 4 National Food and Veterinary Risk Assessment Institute, J. Kairiūkščio 10, LT08409 Vilnius, Lithuania

**Keywords:** ASF, matched, case-control, risk factor, domestic pig, outbreak, veterinary inspection

## Abstract

**Simple Summary:**

In Lithuania, African swine fever (ASF) notifications in domestic pigs and wild boar have been increasing since the entry of ASF from 2014 to 2018. The disease caused serious economic losses and affected commercial and non-commercial pig holdings. We conducted a prospective matched case-control study to investigate the potential risk factors associated with ASF outbreaks in pig farms in Lithuania in 2019. The study revealed that the risk of ASF occurrence in a pig farm was significantly lower if the farm had been inspected by veterinary inspectors, while the risk increased if certain activities, possibly related to the indirect transmission of ASF to a pig farm, were practiced on a farm, e.g., mushroom picking, sharing equipment, etc. Veterinary inspection seemed to increase the level of biosecurity and ASF awareness on a farm, which may have helped to prevent disease introduction. These findings can be used to improve the control and prevention of ASF in domestic pigs in countries affected by ASF.

**Abstract:**

While numerous risk factors of African swine fever (ASF) transmission to domestic pigs have been described, ASF virus introduction has often not been traced back to one single defined cause. The large number of ASF outbreaks that occurred in domestic pigs in Lithuania from 2014 through to 2018 raised the question regarding whether outbreak-specific risk factors and transmission routes could be identified. Therefore, a prospective matched case-control study was designed. Data from 18 outbreaks that occurred in Lithuanian in 2019 and 36 control farms were analyzed. Conditional multivariable logistic regression showed that two or more visits by veterinary inspection of a farm had a significant preventive effect on the occurrence of ASF on a farm (Odds ratio (OR) 14.21, confidence interval (CI) 1.09–185.60 for farms not inspected vs. farms inspected twice or more a year), while certain practices (e.g., mushroom picking, sharing equipment, etc.), which might facilitate the indirect introduction of ASF from fields and forests into piggeries, significantly increased the odds of an outbreak (OR 5.18, CI 1.10–24.44). The results of the study highlight the importance of veterinary inspections for increasing the biosecurity level on pig farms and the awareness of ASF. The knowledge on potential protective and risk factors may help to improve the prevention and control of ASF outbreaks in domestic pig farms in Lithuania and other affected countries.

## 1. Introduction

Lithuania reported the first cases of African swine fever (ASF) in wild boar in January 2014 and was, therefore, the first EU Member State affected by the current ASF epidemic originating from Georgia in 2007 [1]. In July 2014, the first ASF outbreaks emerged in Lithuanian domestic pig farms. With 141 reported ASF outbreaks in domestic pig farms until early 2020, Lithuania has to be considered the most severely affected Baltic state [2]. Although the majority of affected farms kept less than 50 pigs, big industrial farms also suffered from ASF outbreaks with thousands of swine culled [3]. In addition, ASF outbreaks in domestic pigs resulted in trade restrictions within the EU according to the Implementing Regulation (EU) 2021/605 and with third countries, thus impairing the economy of the pig production sector in the country as a minimum. Consequently, it is of utmost importance to reach greater clarity regarding risk factors that may increase the probability of an ASF outbreak in non-commercial or commercial domestic pig holdings.

Several hypotheses exist regarding risk factors of ASF virus (ASFV) introduction into domestic pig holdings. Most studies revealed the circulation of ASFV within the surrounding wild boar population as a considerable risk factor [4,5,6,7]. In Sardinia, the combination of estimated wild boar density and altitude, which indicates the existence of free ranging pigs, was found to be a significant risk factor for ASF [8,9]. Similarly, in Romania, it was found that wild boar abundance around the farm was a risk factor for ASF occurrence on farms, but many other factors may also play a role in this country [7]. In Russia, Vergne et al. [10] found that the presence of ASF-infected wild boar near domestic pig holdings only played a marginal role in disease introduction. In contrast, they stated that outbreaks in domestic pig farms posed a risk to introduce ASF into the surrounding wild boar population. Yet, the authors identified outbreaks in neighboring pig holdings as an important risk factor for disease introduction into a farm, particularly if the level of biosecurity was low [10]. The latter finding is in accord with the results of other studies, in which insufficient biosecurity measures were also found to play a major role in ASF outbreaks [4,5,6,7,11]. Swill feeding, although usually prohibited, was often considered as a risk factor for virus introduction [6,12,13]. This applied particularly to small, private pig holdings (backyard farms) [7,11,14,15,16,17,18]. Despite the dominance of defined risk factors, such as low biosecurity and ASFV circulation within the local wild boar population, it was rarely possible to trace back ASFV introduction in specific outbreak situations to one single defined cause.

The relatively large number of ASF outbreaks that continuously occurred in domestic pigs in Lithuania raised the question around whether any specific risk factors that led to these outbreaks could be identified. We, therefore, conducted a case-control study, matched on location and farm size, to identify potential risk-factors for an increased probability of the occurrence of an ASF outbreak in domestic pig farms, in addition to farm size and the presence of wild boar cases in the vicinity. These potential risk factors may inform policy makers and support disease prevention and control in areas, where ASF is endemic in the wild boar population.

## 2. Materials and Methods

### 2.1. Study Design and Data Collection

A prospective matched case-control-study of ASF outbreaks in domestic pig farms in Lithuania was designed, using the year 2019 as the study period. The study was conducted in collaboration with the State Food and Veterinary Service of the Republic of Lithuania and its territorial divisions. Only farms with 100 pigs or less were included in the study. For each ASF outbreak farm, two control farms were selected. To improve the efficiency of confounding control, cases and controls were matched by location, herd size, and type of farming (commercial vs. non-commercial). In order to adjust the exposure from environment (e.g., presence of ASF in wild boar in the area) for cases and controls, the maximum distance between case and control farms was defined to 25 km. The herd sizes were categorized into two classes, i.e., 1–10 pigs and 11–100 pigs. In Lithuania, a pig farm is regarded as a non-commercial farm (NCF), if it holds up to 10 pigs only for fattening and for own consumption, whereas a commercial pig farm (CF) meets at least one of the following criteria: holds more than 10 pigs, has one or more sows or boars for breeding, or keeps pigs for sale. It was assumed that a small CF with up to 100 pigs has a similar biosecurity level and management behavior as NCF, since the same main biosecurity requirements are compulsory for both NCF and CF in Lithuania. The additional requirements for CF are related to the documentation of activities on a farm, registering visitors, documented biosecurity procedures, and the requirements to provide shower facilities and disinfect the wheels of vehicles entering the farm premises.

Only primary ASF outbreaks were included, while secondary outbreaks were excluded from the analysis because risk factors for primary outbreaks may differ from those relevant for secondary outbreaks. Information on the occurrence of an outbreak, as well as on whether it was considered a primary or a secondary outbreak, was obtained from the Emergency Response Division of the State Food and Veterinary Service of the Republic of Lithuania. All outbreaks were confirmed by the National Food and Veterinary Risk Assessment Institute, which is the Lithuanian National Reference Laboratory for ASF diagnostics. Laboratory testing followed the ASF diagnostic manual [19]. All case farms were visited in person. After receiving an outbreak notification and before visiting the outbreak farm, information on address, coordinates, the number and category of pigs kept on the outbreak farm, and on neighboring farms for the selection of control farms were obtained from the central database of the Farmed Animal Registry of Agricultural Information and Rural Business Centre.

For the selection of control farms, the distances from each case farm to the neighboring farms, categorized in the same farm size group and farm type, were measured using coordinates and addresses using the function ‘Length’ in the menu ‘Measure’ on www.maps.lt (last accessed 11 October 2019). The two farms matching the required criteria and closest to the case farm were chosen as control farms. To reduce the risk of control farms changing specific management or biosecurity procedures after they were contacted with the request to participate in the study, the selected control farms were contacted by phone with a request only on the same day, when veterinary staff were going to visit these farms. If an ASF outbreak occurred in a control farm during the study period, the farm was subsequently considered as a case farm and new controls were selected as described above.

Data on risk factors were collected from outbreak and control farms using a questionnaire, which was an abbreviated version of the official questionnaire for ASF outbreak investigations in Lithuania, omitting only details relating to official control measures. The questionnaire covered the following information: herd information, other animals on a farm and its management, rodent control, cleaning and disinfection, pig feeding, purchase and sales of pigs, visitors and vehicles, other indirect contacts, veterinary inspections of farms, biosecurity, etc. (the full questionnaire is provided in the Supplementary Materials, Document S1).

Staff of the case and control farms were interviewed face-to-face during farm visits by one of two authors, who were veterinarians from the Emergency Response Department. All farm staff of the control farms answered the questionnaire voluntarily. Staff of case and control farms participated voluntarily in the study and gave their oral consent to the anonymous publication of study results. The two veterinarians had official responsibilities to participate in the official outbreak investigations of the case farms. Case farms were visited as soon as possible after the ASF outbreak confirmation, mostly on the day of culling or the day before. Farm owners were the main source of information, but farm workers and family members involved in pig management were also interviewed, if available. Moreover, information collected by the local veterinary authorities during the sampling on farms under ASF suspicion was also used for the epidemiological investigations. In control farms, visual inspections were carried out on as on case farms, but pig pens were not entered to avoid accidental introduction of ASF. During the interviews, answers were recorded on paper and data were later entered into a purpose-built Microsoft Excel file. Data were validated by two persons, who double-checked the entries, before they were used for statistical analyses.

### 2.2. Statistical Analysis

From the questionnaire, 89 variables were generated. Some of the variables were used as phrased in the questionnaire; others were derived by summarizing information from variable(s) of interest. Data were examined for plausibility using box–whisker plots and minimum and maximum values.

The evaluation of the biosecurity on farms was carried out by assessing five biosecurity elements (Supplementary Materials, Document S1, question 26). Each of the five elements were assessed separately and then overall assessment of biosecurity was evaluated (variable Farm biosecurity, Table 1). If one or more elements were assessed risky, then the overall assessment of biosecurity was also risky. Moreover, a variable ‘Sum of detected biosecurity deficiencies on farm’ was created to summarize how many biosecurity elements were evaluated as deficient.

The ‘Reviewer’s estimated reliability score of farm biosecurity’ (Supplementary Materials, Document S1, question 26) variable was based on a 10-point scale estimation of the reliably regarding the installation of biosecurity equipment; the proper and regular use of disinfection mats, rubber boots, and working clothes; and their appropriateness and maintenance. This variable also included an arbitrary assessment on the level on which the farm fulfilled biosecurity requirements. The ‘Veterinary control frequency’ (Supplementary Materials, Document S1, question 17) of pig farms variable was obtained from the official data on farm inspections. During 2019, official veterinarians visited all pig farms that had entries in the national registry; the absolute majority of farms were visited in February–June. Pig farms were not visited by official veterinarians if they were not registered in the database at the time of the inspections or if the farm had no pigs during that time. The ‘Other contacts’ variable was used directly from the questionnaire containing information collected on habits and routine pig management activities, which may represent or increase risks for indirect ASF introduction. The variable indicated that, in the 4 weeks prior to the visit, any of the following actions were conducted: collecting snails, berries, or mushrooms in the fields or forests; working in a forest; sharing vehicles between farms; and directly placing other animals into an indoor space where pigs were kept.

Potential risk factor variables were analyzed for associations with the ASF outbreak status (case/control) as a binary outcome, using univariable conditional logistic regression analysis for categorical variables. The Wilcoxon rank-sum test was used for continuous variables. Variables with *p*-values of less than 0.05 in the univariable analysis were considered for inclusion in a multivariable model.

Conditional multivariable logistic regression was used, due to the matched case control design. Collinearity was assessed for all initially selected variables. Associations between risk factors were considered as relevant, if the correlation coefficient was ≥0.7. In case of collinearity, the variable was selected that was biologically more plausible or summarized more information. A manual step-wise forward selection of variables was adopted to retain the variables that did not exceed *p* > 0.05. Each removed variable was checked for confounding effects and retained in the model, if its exclusion changed the regression coefficient of any other variable by more than 20% [20,21].

Odds ratios of the disease in the presence of the risk factor of interest, together with their 95% confidence intervals (95% CI), were calculated from the regression coefficients, using the function ‘clogit’ in the survival package of R [22]. Pseudo R-squared and Akaike information criterion (AIC) values were used to assess the quality and the model fit of the multivariable conditional logistic regression model. The Pseudo R-squared was estimated using the pscl package with pR2 function [23]. The significance level was set at *p* = 0.05. All calculations were completed using the software package R [24].

## 3. Results

**Figure 1:**
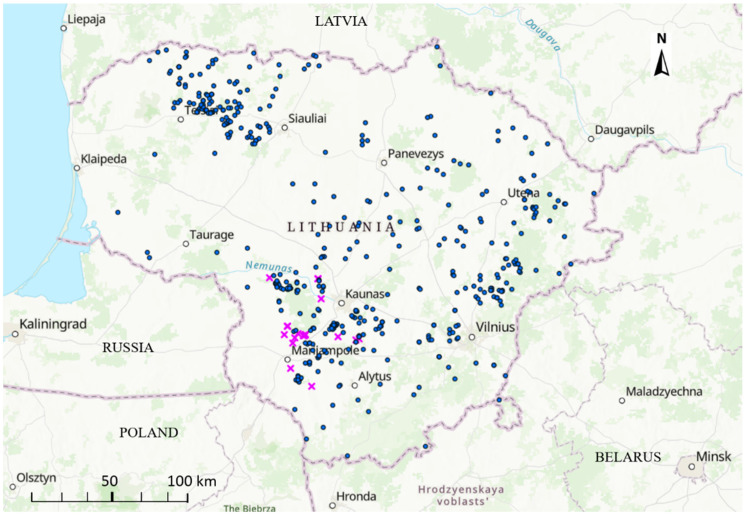
Spatial distribution of African swine fever outbreaks in domestic pigs (pink crosses) and wild boars (blue circles) in Lithuania in 2019.

The first ASF outbreak in Lithuania in 2019 occurred on 6 June and the last one on 11 October 2019. Thus, in total, 19 ASF outbreaks in pig farms were reported in Lithuania in 2019. The outbreaks clustered in the southeastern region of the country (Figure 1). 

All outbreaks occurred in farms with less than 100 animals. With the exception of one secondary outbreak, the remaining 18 were included in the study as case farms (primary outbreaks) and 36 control farms selected. For all case farms, it was the first time they had ASF outbreaks. Descriptive information of case and control farms are presented in Table 2. All contacted control farms kindly agreed to participate in the study.

During the study period, one NCF control farm (no. 6.2) experienced an ASF outbreak and became a case farm (no. 11). Therefore, a new control farm for case no. 6 was selected. For case no. 10, which was a CF with 10 pigs, no other CF with up to 10 pigs was located within a 25-km radius. Therefore, two NCFs with six and seven pigs were selected. For the same reason, for CF case no. 19, one of the two control farms was an NCF. For case no. 14, which was NCF, a small CF with eight pigs was selected as one of the two control farms, due to a lack of sufficient NCF in the defined distance (25 km).

Fourteen case farms and thirty control farms were fattening farms; all others were mixed herds with fattening pigs and other pigs (sows, boars, or piglets). Pigs were kept indoors in all farms.

With the exception of two control farms, the questionnaire and visual farm inspection were conducted on the same day, when they were contacted. The remaining two control farms were visited the day after they were contacted.

The median time between the visit of case farms and the visit to their respective control farms was 5 days (1st Quartile: 1; 3rd Quartile: 7.5 days). The interview of a control farm lasted approximately 90 min, while it took up to 3.5 h to interview a case farm, because the complete official epidemiological investigation was performed at the same time. The median distance between the case and control farms was 2.03 km (min: 0.14 km, max: 24.25 km). There was no statistically significant difference between the distances between the case and control farms to the nearest ASF positive wild boar (4.7 km and 5.5 km, respectively). The median size of the case and control farms was 3 and 2.5 pigs, respectively, and there were 1 to 54 and 49 pigs kept, respectively (Table 2).

In addition to pigs, all case and control farms kept one or more other animal species on their premises (mostly dogs, poultry, cats, cattle, and sheep). Farms where cattle, horses, or sheep were kept reported daily movements from the farm area to nearby fields for management of these animals. The majority of case (15) and control (24) farms had at least one visitor (mostly relatives) during the 4 weeks before the outbreak, respectively. In most cases, relatives came to help with farm work. There was only one visit by a professional, i.e., a private veterinarian, who entered the pig pens for castration of piglets on a commercial control farm.

On two case and two control farms, the farmers or farm workers were also hunters. Within 4 weeks before the visit, three farms (all small CF) sold the pigs. Swill feeding was practiced on three case farms and nine control farms. Potatoes were fed on 8 case farms and 21 control farms. Grass was fed to pigs on five case farms and nine control farms. None of these factors were statistically significantly associated with the occurrence of ASF in the univariable analysis.

Four case and two control farms were not visited by veterinary inspectors in 2019, before they got involved in the case-control study. Two of the four case farms had never been inspected before, because these farms had no record in the official register of pig keepers. The remaining two case farms and the two control farms were not inspected, because there were no pigs registered in these farms from January to June 2019, but these farms had previously been checked. All non-inspected farms had biosecurity deficiencies detected in this study (e.g., absence of a fence around the pig holding, absence of disinfection barriers, clothes and working shoes were not changed when entering the area, where the pigs were kept, etc.).

Univariable analysis identified 19 variables that showed tendencies (*p* < 0.2) in their associations with the occurrence of ASF outbreaks on farms (Table 1). However, due to the small sample size, only six variables with *p* ≤ 0.05 were available for the multivariable analysis. Four of these were related to biosecurity issues: reviewer’s estimated median of reliability score of farm biosecurity, quality and use of disinfection barriers, overall evaluation of farm biosecurity, and the sum of detected biosecurity deficiencies. Due to strong correlations among the four variables describing the quality of biosecurity, only the ‘sum of detected biosecurity deficiencies’ variable was subsequently included in the multivariable model. The other two variables were ‘Veterinary control frequency’ and ‘Other contacts’. In the multivariable conditional logistic regression model, only the variables ‘Veterinary control frequency’ and ‘Other contacts’ were retained as statistically significant (Table 3).

The AIC of the final model was 32.84 (in comparison to 39.55 for the null model) and the pseudo R squares were in the range between 0.5833 (Mc Fadden) to 0.9011 (Cragg and Uhler’s pseudo R square).

## 4. Discussion

The current epidemic, caused by an ASFV genotype II, started in Georgia in 2007. Since then, it has continuously spread and affected more and more countries [25]. In Lithuania, ASF notifications in domestic pigs and wild boars increased since the ASF emerged in the country in 2014 and the disease caused serious economic losses. The continuous spread and wide distribution over several continents require the identification of specific risk factors responsible for ASF outbreaks in domestic pigs. Although the case-control design is known as one of the most efficient choices for evaluating exposure-outcome associations [26,27], only a single case-control study regarding ASF and its introduction into domestic pig holdings within the European setting has so far been published according to our knowledge. This study has been conducted in Romania, where the epidemiological situation, which is characterized by a large number of outbreaks in domestic pigs and only few cases in wild boars, is hardly comparable to the situation in Lithuania [7]. Since it is usually difficult to identify the route of ASFV introduction into a domestic pig farm unambiguously, the current case-control study was performed. The aim was to determine potential risk factors for the introduction of ASF into domestic pig farms. The results might be used to optimize prevention and control of ASF in domestic pigs in Baltic countries, while other affected countries or countries were at risk of ASFV introduction.

For the present prospective case control study, it was planned to include all domestic pig farms in Lithuania that suffered an ASF outbreak in 2019. Thus, no power analysis was conducted prior to the study. In order to increase the power of the study, two control farms for each case farm were selected. When the study was designed, the true number of outbreaks was not yet known. In the interest of feasibility, the number of control farms was limited to two, due to logistical reasons (visits of control farms shortly after outbreaks to avoid that management practices had changed until the farms were visited). These considerations outweighed the expected gain in power that could be obtained with more than two controls. Infected wild boar in the close vicinity of affected domestic pig holdings constitute a considerable risk for ASF introduction into domestic pig holdings [4,5,6,7]. To ensure comparable conditions between case and control farms regarding environmental ASF infection pressure, the control farms were matched to the case farms with respect to their locations. In addition, the control farms were matched for farm size. This approach allowed exploring risk factors not confounded by proximity and density of ASF-affected wild boar and farm size, and improved the efficiency of confounding control.

Due to a clear dominance of ASF outbreaks in small pig holdings (111 outbreaks in farms up to 100 pigs vs. 8 outbreaks in larger farms) from 2014 through to 2018 (data from EU Animal Disease Notification System), the study design intended to include only farms with less than 100 pigs. Based on experience from previous years, at the beginning of the study, it was assumed that approximately 70–100 outbreaks would occur in 2019. However, the true number of outbreaks in the study year was considerably lower, which limited the detection of risk factors that had only little effect. The reasons for the lower number of outbreaks in 2019 is unknown and may need further investigation. The small number of cases, which also led to a small number of controls, resulted in a limited number of variables that could be included in the multivariable analysis. Thus, a manual forward selection to incorporate the most significant factors identified in the univariable analysis was used. Considering the large number of variables in the study and the small number of farms, only variables with a significance level (*p*) of less than 0.05 were considered for building the multivariable model.

By choosing a prospective study design and conducting prompt farms investigations, which were ideally performed at similar time intervals in case and control farms, we tried to minimize the risk of recall bias. In particular, biosecurity measures could be evaluated with a high level of reliability at the time of the outbreaks, so that post-hoc modifications or bias regarding these measures was unlikely. To avoid any quick adaptation in biosecurity measures in control farms after an outbreak in the neighborhood, control farms were only approached and contacted with the request for participation by phone on the way to the farm, i.e., shortly before the visit.

The interviews on the case and control farms were conducted by two persons only and always followed the same standard procedure. Although subjective judgements could not be completely excluded, in particular regarding the summarizing assessments on farm biosecurity, bias was reduced as much as possible. However, the lack of blinding in the study also constitutes a potential bias. The knowledge about the ASF status of the participating farm can affect the farmer’s behavior and also the assessment by the interviewer, thus biasing the outcomes of the questionnaire and the farm evaluation. Due to the prospective character of the study and the legally required epidemiological outbreak investigations (the results of which were used in the study), blinding the interviewers was unfortunately not possible. As the farmers were obviously aware of the ASF status of their pigs, blinding the farms was impossible. However, the interviewers were aware of this potential bias and they tried to be as objective as possible. The results of the present study yielded a significant association between the introduction of ASFV into a pig farm and the variables ‘Veterinary control frequency’ of pig farms and ‘Other contacts’. The odds of an ASF outbreak were found to increase if a pig farm was not visited, or visited only once, by a veterinary inspector. These findings are not surprising, as it can be assumed that such visits increase the awareness of farmers regarding ASF and the risks of becoming affected by the disease. Official veterinarians may also point out biosecurity gaps and demand that the farms are closed. Since the inspectors checked the implementation of biosecurity, at least of mandatory measures, farms with less frequent farm inspections may apply lower biosecurity standards. It is known that biosecurity measures and awareness of farm workers are crucial for the prevention of ASFV introduction [28,29,30]. In a previous survey, farmers mentioned insufficient information as a reason not to invest in biosecurity measures, while veterinarians were named as the main source of information regarding biosecurity [31].

The variable ‘Other contacts’ in the final multivariable model contained several well-known risky practices, which can potentially increase the risk of ASFV incursion into a pig farm, e.g., collecting snails, berries, mushrooms in fields or forests; working in forests; introducing other animals into a pig pen or space in the previous 4 weeks; and sharing and using hired tractors on the farm. The risk factors identified in the analysis are in accord with the current knowledge on risk factors for ASF transmission to domestic pigs. Many risk factors have so far been identified, including the introduction of infected pigs, swill feeding, indirect or direct contact with wild boar, feeding fresh grass or crops, the use of contaminated straw, the presence of infected farms in the neighborhood, proximity to ASF-affected wild boar, density of ASF cases in wild boar, visits by veterinarians and other visitors, low levels of biosecurity, and herd size [4,6,7,8,10,32,33]. In Romania, the proximity to other ASF outbreaks in domestic pig farms appeared to be a strong risk factor. Due to the matching design of the present study, proximity to other ASF outbreaks could not be evaluated. However, there might be other factors which differ between Romania and Lithuania, which could explain the difference in the numbers of affected farms (e.g., less distribution of pig meat from NCF to other pig holders in Lithuania) or the lower pig farm density in Lithuania compared to Romania. However, data from Lithuanian ASF outbreak investigations in 2018 also failed to provide evidence that the ASF status in neighboring pig farms and swill feeding had an impact on the occurrence of new ASF outbreaks (unpublished data of the State Food and Veterinary Service of the Republic of Lithuania). Despite the reduced power of the study, the results correspond to recent studies, where regular and standardized biosecurity controls were suggested to limit ASF introduction and spread [34,35].

Prior to 2019, most areas of Lithuania were affected by ASF and the disease had become endemic in the Lithuanian wild boar population [36]. Only a few small areas in western Lithuania were still free from ASF. Over time, the disease spread westwards, where numerous NCFs were still present. Therefore, the observed spatial distribution of ASF outbreaks in domestic pigs in south-east Lithuania in 2019 was unexpected. Further investigations which consider socio-economic factors, the quality and quantity of veterinary inspections, and ASF wild boar data across the country are necessary to clarify the occurrence of outbreaks.

## 5. Conclusions

This study evaluated potential risk factors associated with outbreaks of ASF in domestic pig farms in Lithuania. The results highlight the importance of veterinary inspections for increasing the biosecurity level on pig farms and ASF awareness. The study also identified risky practices and habits in pig farm management, mainly related to biosecurity measures that are associated with increased indirect transmission of ASF virus to pig farms in Lithuania. The present findings can be used to improve prevention and control of ASF in domestic pigs in Lithuania and in other countries affected by ASF.

## Figures and Tables

**Table 1:** Farm-level variables with *p*-values less than 0.2 in the univariable analysis of African swine fever in a case-control study of domestic pigs in Lithuania in 2019.

	Variable	Cases (*n* = 18)	Controls (*n* = 36)	*p*-Value
1 BioReliability	Reviewer’s estimated of reliability score of farm biosecurity on 10-point scale, (quartiles Q1 and Q3)	7 (5.2/7)	8 (7/9)	0.007
2 RiskBioDesinf	Quality and use of disinfection barriers, changing shoes and clothes (appropriate/not appropriate)	13/5	11/25	0.010
3 OffContrFreq	Veterinary control frequency of pig farms during the study (in 2019) 1 no visit 2 once a year 3 twice and more a year	4 8 6	2 28 6	0.026
4 Other contacts	Collecting snails, berries, and mushrooms in fields/forests; working in forests; introducing other animals into a pig pen or space in the last 4 weeks; sharing and using hired tractors on the farm	12/6	11/25	0.027
5 RiskBioYN	Farm biosecurity has any deficiency (yes/no)	16/2	20/16	0.031
6 RiskBioSumall	Sum (median) of detected biosecurity deficiencies on farm (out of 5)	2	1	0.038
7 PoultryPresent	Poultry is present on farm yes/no	11/7	30/6	0.065
8 FeedStBuilding	Feed is stored in a separate building and one has to bring it from outside to pigs, yes/no	8/10	7/29	0.088
9 OffVetContr	Farm was visited by veterinary inspector for inspection of biosecurity from beginning of 2019 to the end of the study (yes/no)	14/4	34/2	0.095
10 RiskBioSum	Categories of biosecurity risks 0: no deficiencies 1: one 2: two 3: three 4: four 5: all fail	2 6 3 6 0 1	16 8 5 3 3 1	0.102
11 GrindingInside	Grain for feed is ground in pig keeping building	1/17	8/28	0.117
12 BedStWithPigs	Bedding is stored in the same space as pigs	3/15	1/35	0.121
13 FeedStWithPigs	Feed is stored in the same space as pigs	3/15	1/35	0.121
14 RiskBioFomites	Use of tools, equipment, etc. cause risk due to lack biosecurity, yes/no	8/10	8/28	0.127
15 FeedStSepBioBad	Feed is stored in a separate building than pigs and biosecurity of it is improper	2/16	11/25	0.135
16 FeedHome	Pigs are fed only with on farm grown feed	10/8	27/9	0.147
17 DogsPresent	Dog(s) is present on farm	13/5	32/4	0.151
18 RodentControl	Rodent control on farm is implemented	13/5	32/4	0.151
19 FeedStorage	Place of pig feed storage: In the same building as pigs in the same room as pigs in outside building in separate building with good biosecurity separate building with bad biosecurity	2 3 8 4 2	6 1 8 11 12	0.187

**Table 2:** Information of case and control farms given in the questionnaire within the framework of the case-control study on the risk of African swine fever introduction to pig farms in Lithuania in 2019.

Subject	Case Farms (*n* = 18)	Control Farms (*n* = 36)
Number of commercial/non-commercial farms	4/14	6/30
Total number of pigs kept in farms	159	249
Minimum/median/maximum number of pigs per farm	1/3/54	1/2.5/49
Minimum/median/maximum number of pigs in commercial farms	6/31/54	8/30.5//49
Minimum/median/maximum number of pigs in non-commercial farms	1/2.5/6	1/2/7
Median distance (km) (minimum–maximum) from study farms to the closest officially registered ASF positive wild boar found before the visit	4.7 (0.88–9.27)	5.5 (1.16–30.18)
Median age (minimum–maximum) of farm owners	64 (44–81)	63 (29–83)

**Table 3:** Results of the multivariable conditional logistic regression model in a matched case-control study on African swine fever in domestic pigs in Lithuania in 2019.

Parameter	Levels	Coefficient	OR ^1^	Lower 95% Confidence Interval	Upper 95% Confidence Interval	*p*-Value
Veterinary control frequency	Twice or more/year	Reference				
	Once/year	1.41	4.10	0.62	26.98	0.1418
	Not at all	2.65	14.21	1.09	185.60	0.0429
Other contacts ^2^	No	Reference				
	Yes	1.65	5.18	1.10	24.44	0.0375

^1^ OR: odds ratio. ^2^ Other contacts: collecting snails, berries, mushrooms in fields/forests; working in forests, introducing other animals into a pig area/space in the last 4 weeks; sharing and using hired vehicles).

## Data Availability

The data used in the analyses can be obtained from the author by request.

## Supplementary Materials

The following is available online at https://www.mdpi.com/article/10.3390/ani12010115/s1, Document S1: Questionnaire for epidemiological investigation of ASF outbreak
