# Supplementary material for: African Swine Fever Outbreaks in Lithuanian Domestic Pigs in 2019
Source: Animals (Basel). 2022 Jan 4;12(1):115. doi: 10.3390/ani12010115 (PMC8749716; doi:10.3390/ani12010115)
Supplement: Supplementary file 1 [file animals-12-00115-s001.zip › animals-1366090-supplementary/Supplementary Material Document S1 questionnaire.pdf]

Document S1. Questionnaire for epidemiological investigations of an African swine fever outbreak

QUESTIONNAIR FOR EPIDEMIOLOGICAL INVESTIGATION ON CASE AND CONTROL FARMS

(sections of the official ASF-outbreak questionnaire, which were not relevant for the present case control study, were omitted)

Date of visit: Name of interviewer:

Case farm identification No: Address:

Control farm identification No:

I. INFORMATION OF THE HOLDING

1. General information:

Owner of the holding: legal entity □ natural person □

____

(Name, surname/company name, address)

Contact phone no.:______________

Veterinary confirmation No: _______________

Name(s) and responsibility of interviewed person:____________________

Control farm: Location

Geographic coordinates:

Straight distance (km, one decimal number) to the ASF case farm: _____________

Type of pig farming: commercial □ non-commercial □

2. Herd type:

□ Breeding herd

□ Fattening herd

□ Herd of other purpose (specialized company)

□ Sows before farrowing

□ Other (.......................................)

□ Mixed-breed herd

□ Piglet farming herd

□ Farrowing herd

□ Reproductive herd

4. Swine categories and numbers:

Category	Number of pigs
Piglets up to 3 months	
Pigs from 3 to 8 months	
Pigs over 8 months	

Sows (replacement, major, pregnant, not pregnant)	
Boars	
**Total**	

5. Pig holding is registered in the official data base Yes □ No □

7. Are other animals present on the farm (indicate number for each species): Yes □ No □ ;

if "Yes" what animals:

□ bovines ______________ □ sheep ____________

□ goats____________ □ poultry ____________

□ dogs ____________ □ cats ____________

□ horses_______ □ other ____________ (specify)

- The other animals leave farm territory (Indicate species)_____________ Yes □ No □
- Other animals are kept together in the same premise (the same room/space) (Indicate species)__________________ Yes □ No □
- Is there a physical barrier (solid walls without holes, gaps and etc.) between pigs and other animals in the building? Yes □ No □
- Can pigs and other animals have direct contact with each other Yes □ No □
- Can pigs and animals have indirect contact with each other Yes □ No □
- Additional information____

7.1 Movement of animals from pasture/forest to farm in the last 4 weeks Yes □ No □

If yes, which species and purpose___________________________

Every day □

Several times per week □

Once per week and less □

8. Population of rodent (mice, rats):

Ongoing rodent control □ Yes □ No

- is done by specialized company □ Yes □ No

- is done by owners □ Yes □ No

- high (visible problems with rodents) □

- average (no problems, only signs of rodents) □

- low (rodents and their signs are not observed) □

Additional information____________________________

9. Cleaning, washing of premises:

almost exclusively dry cleaning □

washing without using high-pressure water □

washing in cold or hot high-pressure water without disinfectants □

Cleaning is carried out:

periodically (how frequently) daily □
by taking out from the individual or group holdings □
on the "all-in-all-out" basis □
not carried out □
in presence of animals □

Disinfection is carried out:

periodically (how frequently) ______________________________ □
by taking out from the individual or group holdings □
on the "all-in-all-out" basis □
not carried out □
in presence of animals □

10. Ventilation systems:

natural ventilation □
mechanical ventilation □

separate systems by sections of premise	Yes □	No □
filters	Yes □	No □
insect nets are installed	Yes □	No □

Additional information about protection of windows, doors and ventilation openings from insects _______
____

11. Collection and storage (before dispatch for utilization) of dead animal in the last 4 weeks:

Yes □ No □

If Yes

In closed leakage prove containers □

□ inside a barn or utility room
□ on the premise territory
□ on the edge of premise territory

Not in containers, openly □

□ inside a barn or utility room
□ on the premise territory
□ on the edge of premise territory

12. Pig feeding:

□ fed only non-animal origin feed produced by themselves and purchased
□ fed only non-animal origin feed produced by themselves
□ fed only non-animal origin feed which is purchased
□ green fodder is used (grass, potatoes and etc)
□ fed with purchased feed of animal origin (e. g.: blood, fish meal and etc.)

□ fed with milk of cows (goats, sheep) from own farm

□ feeding with milk processing products or by products from enterprises

□ other feeding practices________________________________

12.1 In-depth information on feeding (diet, technology, feed suppliers):

Whole-meal from own grown grain □ Is it steamed Yes □ No □

Bought whole-meal □ Is it steamed Yes □ No □

Comments____

12.2 Feeding with food waste from canteens or own household kitchen: □ Yes □ No

if "Yes", is it:

□ heat-treated □ not heat treated

12.5 Feed storage:

Specify whether feed and bedding are protected from wild boars □ Yes □ No

In the same space/room where pigs are kept □

In the same building next to pig holding space □

In the same pig keeping building, but you have to bring feed from outside the building □

Biosecurity is good □

Biosecurity is bad □

In a separate building with good biosecurity □

In a separate building with bad biosecurity □

Grain is ground in a pig holding space Yes □ No □

Other

Comments ____

12.6 Beddings: □ homemade □ acquired or bought

□ straw □ sawdust □ No bedding

12.7 Storage place of beddings (other information):

In the same space/room where pigs are kept □

In the same building as pigs □

In the same pig keeping building, but you have to bring feed from outside the building □

Another building:

With good biosecurity □

With bad biosecurity □

Additional information____

13. Insemination:

□ natural mating

□ artificial insemination with on the farm produced semen

□ artificial insemination with purchased semen

□ insemination is performed by farmer (no service from outside)

□ other (e.g. mixed)______________________________

15. Animal purchases and sales within the last 4 weeks:

a) purchase: Yes □ No □

Date	Quantity	Age, category	Place of purchase	Herd origin

b) sale: Yes □ No □

Date	Quantity	Age, category	Place of purchase	Herd origin

Movement routes of the purchased pigs:

□ from seller's farm

□ trader's premises

□ auctions /exhibitions/markets

□ shipment directly from the herd of origin by own transport

□ shipment directly from the herd of origin by means of carriers

□ direct shipment with visiting other herds

16. Distance from an outbreak to the nearest pig holdings/herds:

a)

No	Name and address	Distances (km)	If there was a ASF outbreak - distance in km
1			
2			

b) Distance to the nearest positive wild boar hunted or found dead during the last 6 months:

**No**	**Location and coordinates WGS**	**Distances (km)**	**Date**

17. Official veterinary control of the farm

Yes □ No □

If "Yes", then:

□ once a year

□ once during the pig fattening period

□ twice a year

□ more often

Date of last visit ____________

20. Visitors within the last 4 weeks: **Yes □ No □**

a) controlling authorities:

□ veterinary services

□ breeding service

□ environmental protection

□ fire protection

□ National Paying Agency

□ other authorities

b) service providers:

□ inseminators

□ carriers

□ feed suppliers

□ technical staff

□ attending/holding veterinarian:

identification No: ____________________________

address, tel.: ________________________________

c) other persons:

□ animal traders

□ butcher

□ hunter

□ other ______________________

(specify)

The visitor had contact with pigs on the farm Yes □ No □

The visitors entered pig farm/ area only Yes □ No □

(specify which visitors had which contacts and date)________________________________

21. Movement of transport to pig farm area for last 4 weeks Yes □ No □

List of transport that arrived to the farm in the last 4 weeks (dates and purpose)

____

Was there a sharing of farm transport with other farms in the last 4 weeks Yes □ No □

Farm transport is used for work in fields/pastures in the last 4 weeks Yes □ No □

Farm transport is used for work in forests in the last 4 weeks Yes □ No □

Visiting transport on the farm in forests in the last 4 weeks Yes □ No □

Comments if answered yes____

22. Other contacts within the last 4 weeks: **Yes □ No □**

Possible contact with wild boars Yes □ No □____________________________________

Common use of machines or parts of machines (e.g. grass or silage choppers) □ Yes □ No

Sharing of tools/gear with neighbors/friends within the last 4 weeks __________ □ Yes □ No

Other possibilities of the contact:

____

(specify)

22.1 Movement of farm personnel to forest in the last 4 weeks **Yes □** **No □**

Purpose of movement ______________________________

Once per week □

2-3 times per week □

More than 3 times per week □

Date of last visit_________

22.3 Movement of farm personnel to pastures in the last 4 weeks **Yes □** **No □**

Purpose of movement animal care, works on fields

Once per week □

2-3 times per week □

More than 3 times per week □

Date of last visit ___________

22.4 Additional information regarding visitors

□ there is a hunter among the owners/family, who lives/works on the farm

□ there is a hunter among workers who has contact with pigs

□ there is a hunter among other farm workers with no direct contact with pigs

□ hunters among friends, relatives and etc.,

□ no hunters on farm

23. ASF historical data:

a) ASF – was previously detected □ Yes □ No

Date of confirmation: ___________

26. Biosecurity assessment in the holding

	Measure	Comments and observations	Is there a risk?	
			YES	NO
External biosecurity				
4	Fence and gate quality			
5	Manure keeping place is fenced			
6	Disinfectant barriers (quality and use)			
7	Fomites (only for pigs or not)			
8	Appropriate environment (clean and etc)			

How reliably can be concluded that disinfection matts, rubber boots and working clothes for biosecurity purposes are maintained in good conditions, are used properly, regularly and that a farmer follows biosecurity requirements (assessment by the reviewer)

Not reliable	1	2	3	4	5	6	7	8	9	10	Reliable

VII. ADDITIONAL INFORMATION
